# Untold Stories of Grief and Joy in Aging: An Intergenerational Art-Based Project

**DOI:** 10.1093/geroni/igaf122.4228

**Published:** 2025-12-31

**Authors:** Jeannette Sager, Anna Gibbons, Gwendolyn Hoeffgen, Domenic Toliver, Adela Cardona Puerta, Max Goldman, Cal Storrs, Serena Hasworth

**Affiliations:** Portland State University, Portland, Oregon, United States; Portland State University, Portland, Oregon, United States; Portland State University, Portland, Oregon, United States; Portland State University, Portland, Oregon, United States; Portland State University, Portland, Oregon, United States; Portland State University, Portland, Oregon, United States; Portland State University, Portland, Oregon, United States; Portland State University, West Linn, Oregon, United States

## Abstract

Narratives around aging are often reduced to cliches, limiting opportunities to recognize complexity, diversity, and dignity in aging. To address this gap, Portland State University’s GSA Student Chapter organized Untold Stories of Grief and Joy, an arts-based, intergenerational project designed to foster reflection, dialogue, and connection across the lifespan. The project was hosted in an affordable residential community, where eight older adult participants engaged in guided reflection, relationship building, and artistic creation. An interdisciplinary group of student facilitators supported participants in four weekly workshops that featured a new prompt around joy and grief and an art medium (e.g., photography, poetry). Each week, residents shared their artwork and the stories behind them. The project culminated in a community-wide art exhibition where participants displayed their art and shared reflections. The project was evaluated using a focus group with participants, interviews with facilitators, and survey feedback from exhibition attendees. Findings from the evaluation highlighted the project’s impact on community building and intergenerational engagement, with bonds between residents, students, and the broader community. Participants shared how they reframed perspectives on aging, embracing the possibility of new beginnings at every age. The workshops fostered self-exploration, empowerment, vulnerability, healing, and for some, a newfound identity as an artist. Engagement with art, grief and joy, and the aging process provided participants with opportunities to express complex emotions and experiences through creative processes. This project created replicable tools for other GSA Student Chapters to bring this work to long-term care communities, community centers, and other settings.

